# Risk factors for delirium among hospitalized patients in Zambia

**DOI:** 10.1371/journal.pone.0249097

**Published:** 2021-04-08

**Authors:** Justin K. Banerdt, Kondwelani Mateyo, Yan Yan, Dandan Liu, Yi Zuo, Chiara Di Gravio, Julia C. Thome, Elisabeth D. Riviello, Deanna Saylor, E. Wesley Ely, Douglas C. Heimburger

**Affiliations:** 1 Department of Internal Medicine, Yale University School of Medicine, New Haven, Connecticut, United States of America; 2 Vanderbilt University School of Medicine, Nashville, Tennessee, United States of America; 3 University of Zambia School of Medicine, Lusaka, Zambia; 4 University Teaching Hospital, Lusaka, Zambia; 5 Department of Biostatistics, Vanderbilt University Medical Center, Nashville, Tennessee, United States of America; 6 Beth Israel Deaconess Medical Center, Boston, Massachusetts, United States of America; 7 Harvard Medical School, Boston, Massachusetts, United States of America; 8 Johns Hopkins University School of Medicine, Baltimore, Maryland, United States of America; 9 Critical Illness, Brain Dysfunction, and Survivorship (CIBS) Center, Vanderbilt University Medical Center, Nashville, Tennessee, United States of America; 10 Department of Medicine, Vanderbilt University Medical Center, Nashville, Tennessee, United States of America; 11 Tennessee Valley Veteran’s Affairs Geriatric Research Education Clinical Center (GRECC), Nashville, Tennessee, United States of America; 12 Vanderbilt Institute for Global Health, Nashville, Tennessee, United States of America; Clinca Geriatrica, ITALY

## Abstract

**Objective:**

To identify risk factors for delirium among hospitalized patients in Zambia.

**Methods:**

We conducted a prospective cohort study at the University Teaching Hospital in Lusaka, Zambia, from October 2017 to April 2018. We report associations of exposures including sociodemographic and clinical factors with delirium over the first three days of hospital admission, assessed using a modified Brief Confusion Assessment Method (bCAM).

**Findings:**

749 patients were included for analysis (mean age, 42.9 years; 64.8% men; 47.3% with HIV). In individual regression analyses of potential delirium risk factors adjusted for age, sex and education, factors significantly associated with delirium included being divorced/widowed (OR 1.64, 95% CI 1.09–2.47), lowest tercile income (OR 1.58, 95% CI 1.04–2.40), informal employment (OR 1.97, 95% CI 1.25–3.15), untreated HIV infection (OR 2.18, 95% CI 1.21–4.06), unknown HIV status (OR 2.90, 95% CI 1.47–6.16), history of stroke (OR 2.70, 95% CI 1.15–7.19), depression/anxiety (OR 1.52, 95% CI 1.08–2.14), alcohol overuse (OR 1.96, 95% CI 1.39–2.79), sedatives ordered on admission (OR 3.77, 95% CI 1.70–9.54), severity of illness (OR 2.00, 95% CI 1.82–2.22), neurological (OR 7.66, 95% CI 4.90–12.24) and pulmonary-system admission diagnoses (OR 1.91, 95% CI 1.29–2.85), and sepsis (OR 2.44, 95% CI 1.51–4.08). After combining significant risk factors into a multivariable regression analysis, severity of illness, history of stroke, and being divorced/widowed remained predictive of delirium (p<0.05).

**Conclusion:**

Among hospitalized adults at a national referral hospital in Zambia, severity of illness, history of stroke, and being divorced/widowed were independently predictive of delirium. Extension of this work will inform future efforts to prevent, detect, and manage delirium in low- and middle-income countries.

## Introduction

Delirium is a potentially modifiable form of acute neurologic dysfunction that is common among hospitalized patients, with particularly high rates among intensive care unit (ICU) patients [1]. Delirium is an independent predictor of long-term mortality [2,3] as well as cognitive and functional impairment [4,5]. Healthcare costs attributable to delirium have been estimated to range from $143 billion to $152 billion annually in the United States alone [6].

Studies in high-income countries (HICs) have identified both modifiable and non-modifiable risk factors for delirium [1]. Elucidation of these risk factors has informed the development of effective evidence-based strategies for preventing delirium and associated poor clinical outcomes, particularly within the ICU setting [7]. Although delirium is now considered a serious issue of public health importance among hospitalized patients in HICs [7,8], limited information exists about risk factors for delirium in low- and middle-income countries (LMICs).

Studies of delirium conducted in sub-Saharan Africa have had generally not used validated instruments for delirium assessment and have not rigorously examined potential risk factors for delirium in hospitalized patients [9]. One recent prospective study of 160 mechanically ventilated patients in four ICUs in Kampala, Uganda identified several demographic, admission, clinical, and treatment-related risk factors for delirium using a validated tool for delirium assessment [10]. However, the study did not include medical and surgical patients from non-ICU settings, where many critically ill patients are cared for in LMICs with limited ICU capacities [11].

We recently reported that delirium is a strong, independent predictor of 6-month mortality and disability among hospitalized patients in Zambia [12]. The current investigation used the same prospectively collected database to study sociodemographic and clinical risk factors for delirium during the first three days of admission among medical and surgical patients hospitalized at a university teaching hospital in Zambia.

## Materials and methods

### Study design and population

We conducted a prospective cohort study with enrollment occurring between October 30, 2017, through April 5, 2018, at the University Teaching Hospital (UTH), a 1655-bed national referral hospital in Lusaka, Zambia. Patients aged 18 years or older admitted to the medical and surgical wards of UTH were eligible for inclusion in the study. Those who did not understand either English, Nyanja, or Bemba were excluded. In addition, a separate study to examine delirium point prevalence was conducted in the same setting, which enrolled all admitted patients from April 30 through May 6, 2018. Additional details on the study design and methods have been reported elsewhere [12].

The University of Zambia Biomedical Research Ethics Committee, the Zambia National Health Research Authority, and the Vanderbilt University Institutional Review Board (IRB) granted ethical approval. Written informed consent was obtained from participants or their legally authorized representatives prior to enrollment in the study. Capacity for consent was determined by evaluating whether participants could express understanding of the basic aims of the research and what information would be collected from them, demonstrate reasoning about the risks and benefits of being involved in the study, and state a clear preference regarding participation. For participants who lacked capacity for consent (usually due to altered mental status), a legally authorized representative was asked to provide consent.

### Outcome

Delirium assessment was conducted using the modified Brief Confusion Assessment Method (bCAM) [13,14], a validated instrument to assess delirium in acutely ill adults. Nyanja and Bemba language versions of the bCAM were developed through translation and back-translation.

Participants were assessed once daily for delirium during their first three days of hospitalization. The primary outcome, delirium status, was defined as positive if any delirium assessment during the first three days of admission indicated delirium, and negative if at least two assessments were done and no assessment indicated delirium.

### Exposures data collection

Patient sociodemographic and clinical characteristics were collected at study enrollment, which occurred within 24 hours of hospital admission. Sociodemographic data included age, sex, marital status, education, occupation, and monthly income. HIV status on admission as well as HIV duration and antiretroviral therapy where applicable were also recorded. Resource limitations precluded laboratory confirmation of many suspected tuberculosis (TB) cases, so participants were considered to have TB if they received a clinical diagnosis of TB by a physician on admission or were on antituberculosis therapy prior to admission. Medical history was collected on the following conditions by interviewing the participants and accessing their medical charts: hypertension, stroke, depression or anxiety (by self-report), alcohol overuse (defined as greater than five drinks on a single occasion), heart disease, chronic liver disease, chronic lung disease, chronic kidney disease, and cancer.

Physiologic vital sign data and Glasgow Coma Scale were collected at the bedside upon study enrollment and used for calculating severity of illness scores including the Universal Vital Assessment (UVA) [15], the Modified Early Warning Score (MEWS) [16], and the Quick Sequential Organ Failure Assessment (qSOFA) [17]. The UVA score was the primary severity of illness score used. It was derived and validated using 13 cohort studies of adult hospitalized patients from Gabon, Malawi, Sierra Leone, Tanzania, Uganda, and Zambia [15]. The score comprises seven clinical variables: temperature, heart rate, respiratory rate, blood pressure, oxygen saturation, level of consciousness, and HIV serostatus. Scores range from 0 to 13 and can be used to stratify patients into risk categories; for example, as compared to low-risk patients (UVA score 0–1) high risk patients (UVA score > 4) have 10 times higher odds of mortality [15].

Clinical admission diagnoses were recorded, coded into 26 separate diagnoses including sepsis, and used to classify participants according to the organ systems affected (e.g., pneumonia was classified as pulmonary). Data were also collected on mid-upper arm circumference, a marker of nutritional status [18], and whether sedatives (benzodiazepines) were ordered on admission.

REDCap was used for secure entry, storage, and management of all study data [19].

### Statistical analysis

Participants were excluded from analysis if they had only one delirium assessment and it was negative. The remaining cohort was the primary sample used for all risk factor modeling. Descriptive statistics were provided for all exposure variables by delirium status and for the overall cohort.

Logistic regression analyses adjusted for age, sex, and education were conducted to assess associations between individual exposure variables and delirium status; we called these individual regression analyses. Medical history variables and organ system admission diagnosis variables were grouped to avoid potential confounding. The HIV exposure variable was modeled by combining HIV status and antiretroviral therapy status into four levels: HIV uninfected, HIV infected on antiretroviral therapy, HIV infected not on antiretroviral therapy, and unknown HIV status. For categorical exposure variables, reference levels were chosen *a priori* as the prevalent level. The threshold for statistical significance was set *a priori* at p<0.05.

Significant risk factors from the individual regression analyses were then included in a combined multivariable regression analysis to determine which variables remained independently predictive of delirium in the presence of the others. As in the individual regression analyses, the combined multivariable regression analysis controlled for age, sex, and education. We decided *a priori* that if more than one severity of illness score was found to be a significant risk factor in the individual analyses, we would choose one for inclusion in the combined analysis with the following priority orders: UVA, MEWS and qSOFA. We selected UVA because it was validated in six sub-Saharan African countries among 5,573 hospitalized adult patients, including those with HIV and sepsis [15]. Because HIV status is a component of the UVA score, and HIV status was significant in individual analysis, we removed HIV status from the UVA score calculation in the combined analysis; doing so has been shown to have minimal impact on the score’s performance [15].

Analyses were performed using R.

## Results

Among 813 enrolled participants in the primary cohort, 64 were excluded because they had only one delirium assessment and it was negative, leaving a total of 749 who met eligibility criteria for inclusion in the primary analysis (**Table 1**). Separately, 330 participants were enrolled in the seven-day point prevalence study. Delirium prevalence (positive bCAM) at enrollment was 47.0% (95% CI, 43.5%-50.5%) in the primary cohort versus 43.6% in the seven-day point prevalence study. HIV prevalence was 47.3% in the primary cohort. The primary cohort had a mean UVA severity of illness score of 4.73 (SD 3.08), indicating a population with a high risk of death.

**Table 1:** Baseline characteristics of participants by delirium status.

	No Delirium (n = 320)	Any Delirium^a^ (n = 429)	Total (n = 749)^b^
**Age**			
Mean (SD)	39.9 (15.1)	45.2 (17.0)	42.9 (16.4)
**Sex, No. (%)**			
Female	102 (31.9)	162 (37.8)	264 (35.2)
Male	218 (68.1)	267 (62.2)	485 (64.8)
**Admission type, No. (%)**			
Medical admission	196 (61.2)	314 (73.2)	510 (68.1)
Surgical admission	124 (38.8)	115 (26.8)	239 (31.9)
**Marital Status, No. (%)**			
Married/co-habited	186 (58.1)	216 (50.3)	402 (53.7)
Divorced/widowed	58 (18.1)	132 (30.8)	190 (25.4)
Single (never married)	75 (23.4)	76 (17.7)	151 (20.2)
**Education, No. (%)**			
Grade 1–7	94 (29.4)	149 (34.7)	243 (32.4)
Grade 8–12	170 (53.1)	196 (45.7)	366 (48.9)
Never attended	10 (3.1)	22 (5.1)	32 (4.3)
University, college, post-graduate	40 (12.5)	54 (12.6)	94 (12.6)
**Monthly Income (Zambian Kwacha/USD equivalent), No. (%)**			
More than 1500 Kwacha/150 USD	92 (28.8)	88 (20.5)	180 (24.0)
501–1500 Kwacha/50.1–150 USD	77 (24.1)	103 (24.0)	180 (24.0)
0–500 Kwacha/0-50 USD	150 (46.9)	234 (54.5)	384 (51.3)
**Occupation, No. (%)**			
In-wage employment	163 (50.9)	184 (42.9)	347 (46.3)
Farmer/works on own land	27 (8.4)	39 (9.1)	66 (8.8)
Unemployed	90 (28.1)	117 (27.3)	207 (27.6)
Informal employment	39 (12.2)	78 (18.2)	117 (15.6)
**Arm Circumference (cm)**			
Mean (SD)	26.7 (6.56)	26.3 (5.34)	26.5 (5.90)
**HIV status and antiretroviral therapy status, No. (%)**			
HIV-uninfected	160 (50.0)	181 (42.2)	341 (45.5)
HIV-infected with antiretroviral therapy	128 (40.0)	158 (36.8)	286 (38.2)
HIV-infected without antiretroviral therapy	19 (5.9)	42 (9.8)	61 (8.1)
Unknown HIV status	11 (3.4)	41 (9.6)	52 (6.9)
**HIV duration (years, among HIV-infected)**			
Mean (SD)	4.64 (4.86)	3.21 (4.28)	3.84 (4.59)
**TB admission diagnosis**^**c**^**, No. (%)**			
No	237 (74.1)	291 (67.8)	528 (70.5)
Yes	83 (25.9)	138 (32.2)	221 (29.5)
**Ongoing antituberculosis therapy at admission (among participants with TB history), No. (%)**			
No	79 (24.7)	119 (27.7)	198 (26.4)
Yes/Defaulted	36 (11.2)	57 (13.3)	93 (12.4)
**Past Medical History, No. (%)**			
Hypertension	43 (13.4)	97 (22.6)	140 (18.7)
Stroke	7 (2.2)	35 (8.2)	42 (5.6)
Depression/anxiety	167 (52.2)	275 (64.1)	442 (59.0)
Heart disease	19 (5.9)	27 (6.3)	46 (6.1)
Chronic liver disease	9 (2.8)	5 (1.2)	14 (1.9)
Chronic lung disease	8 (2.5)	9 (2.1)	17 (2.3)
Chronic kidney disease	17 (5.3)	26 (6.1)	43 (5.7)
Cancer	3 (0.9)	7 (1.6)	10 (1.3)
Alcohol overuse	113 (35.3)	178 (41.5)	291 (38.9)
**Admission diagnoses organ system category, No. (%)**			
Neurological	47 (14.7)	195 (45.5)	242 (32.3)
Cardiovascular	133 (41.6)	214 (49.9)	347 (46.3)
Pulmonary	86 (26.9)	154 (35.9)	240 (32.0)
Gastrointestinal	82 (25.6)	69 (16.1)	151 (20.2)
Renal	54 (16.9)	86 (20.0)	140 (18.7)
Hepatic	15 (4.7)	25 (5.8)	40 (5.3)
Musculoskeletal	70 (21.9)	49 (11.4)	119 (15.9)
Endocrine	17 (5.3)	30 (7.0)	47 (6.3)
**Sepsis admission diagnosis, No. (%)**	24 (7.5)	74 (17.2)	98 (13.1)
**Sedatives ordered on admission, No. (%)**	7 (2.2)	30 (7.0)	37 (4.9)
**UVA with its HIV variable**			
Mean (SD)	2.73 (2.38)	6.31 (2.63)	4.73 (3.08)
**UVA without its HIV variable**			
Mean (SD)	1.81 (1.91)	5.34 (2.35)	3.79 (2.79)
**MEWS**			
Mean (SD)	3.28 (1.86)	4.29 (2.04)	3.86 (2.02)
**qSOFA, No. (%)**			
0	116 (36.2)	22 (5.1)	138 (18.4)
1	132 (41.2)	156 (36.4)	288 (38.5)
2	68 (21.2)	174 (40.6)	242 (32.3)
3	2 (0.6)	68 (15.9)	70 (9.3)

Abbreviations: HIV, human immunodeficiency virus; TB, tuberculosis; UVA, Universal Vital Assessment; MEWS, Modified Early Warning Score; qSOFA, quick Sequential Organ Failure Assessment.

^a^The outcome variable of interest was any delirium as calculated from the daily bCAM, such that any positive delirium evaluations at any of the timepoints resulted in a positive delirium outcome.

^b^Cell counts for categorical variables may not always sum to n = 749 due to missing values.

^c^Resource limitations precluded laboratory confirmation of suspected TB cases, so participants were considered to have TB if they received a clinical diagnosis of TB by a physician at admission or were on antituberculosis therapy.

### Individual regression analyses of exposure variables

Significant sociodemographic-related risk factors for delirium included being divorced/widowed (OR 1.64, 95% CI 1.09–2.47), having informal employment as an occupation (OR 1·97, 95% CI 1.25–3.15), and being in the lowest tercile of monthly income level (OR 1.58, 95% CI 1.04–2.40) (**Table 2**).

**Table 2:** Individual regression analyses of exposure variables^a^.

	Odds Ratio for Delirium	95% CI	P Value
**Admission type**			
Medical	*Reference*		
Surgical	0.63	(0.43–0.90)	.012
**Marital status**			
Married/cohabiting	*Reference*		
Divorced/widowed	1.64	(1.09–2.47)	.018
Single (never married)	1.17	(0.77–1.79)	.455
**Occupation**			
In-wage employment	*Reference*		
Unemployed	1.01	(0.69–1.47)	.978
Farmer/works on own land	0.90	(0.51–1.62)	.727
Informal employment	1.97	(1.25–3.15)	.004
**Monthly income (Zambian Kwacha/USD equivalent)**			
More than 1500 Kwacha/150 USD	*Reference*		
501–1500 Kwacha/50.1–150 USD	1.40	(0.90–2.17)	.136
0–500 Kwacha/0-50 USD	1.58	(1.04–2.40)	.032
**HIV status and antiretroviral therapy status**			
HIV uninfected	*Reference*		
HIV infected with antiretroviral therapy	1.11	(0.79–1.55)	.544
HIV infected without antiretroviral therapy	2.18	(1.21–4.06)	.011
Unknown HIV status	2.90	(1.47–6.16)	.003
**HIV duration (years)**	0.92	(0.87–0.96)	.001
**TB admission diagnosis**			
Negative	*Reference*		
Positive	1.35	(0.97–1.89)	.075
**Ongoing antituberculosis therapy at admission (among participants with TB history)**			
No	*Reference*		
Yes/defaulted	0.94	(0.53–1.67)	.838
**Medical history**			
Hypertension	1.32	(0.82–2.17)	.257
Stroke	2.70	(1.15–7.19)	.032
Depression/anxiety	1.52	(1.08–2.14)	.017
Alcohol overuse	1.96	(1.39–2.79)	< .001
Heart disease	0.54	(0.27–1.09)	.084
Liver disease	0.42	(0.13–1.28)	.139
Lung disease	0.79	(0.29–2.23)	.649
Kidney disease	1.03	(0.52–2.11)	.924
Cancer	1.30	(0.34–6.30)	.718
**UVA with its HIV variable**	1.76	(1.61–1.92)	< .001
**UVA without its HIV variable**	2.00	(1.82–2.22)	< .001
**MEWS**	1.31	(1.21–1.43)	< .001
**qSOFA**			
1	6.76	(3.96–11.55)	< .001
2	15.12	(8.59–26.63)	< .001
3	197.32	(44.41–876.69)	< .001
**Admission diagnosis organ system category**			
Neurological	7.66	(4.90–12.24)	< .001
Cardiovascular	1.46	(0.98–2.17)	.064
Pulmonary	1.91	(1.29–2.85)	.001
Gastrointestinal	1.09	(0.70–1.70)	.711
Renal	1.38	(0.90–2.12)	.140
Hepatic	1.48	(0.72–3.14)	.291
Musculoskeletal	1.13	(0.67–1.93)	.654
Endocrine	1.52	(0.78–3.04)	.230
**Sepsis admission diagnosis**	2.44	(1.51–4.08)	< .001
**Sedatives ordered on admission**	3.77	(1.70–9.54)	.002
**Mid-upper arm circumference**	0.99	(0.96–1.02)	.459

^a^Regression analyses adjusted for age, sex, and education were conducted to assess associations between individual exposure variables and delirium status. Each bolded variable category indicates a separate regression analysis.

Significant HIV-related risk factors for delirium included having untreated HIV infection (OR 2.18, 95% CI 1.21–4.06) or unknown HIV status (OR 2.90, 95% CI 1.47–6.16) (Table 2); having treated HIV infection was not a significant risk factor for delirium. TB admission diagnosis and antituberculosis therapy were also not significant delirium risk factors. Among people living with HIV, longer duration since HIV diagnosis was significantly protective against delirium (OR 0.92 for every additional year, 95% CI 0.87–0.96).

**Fig 1:**
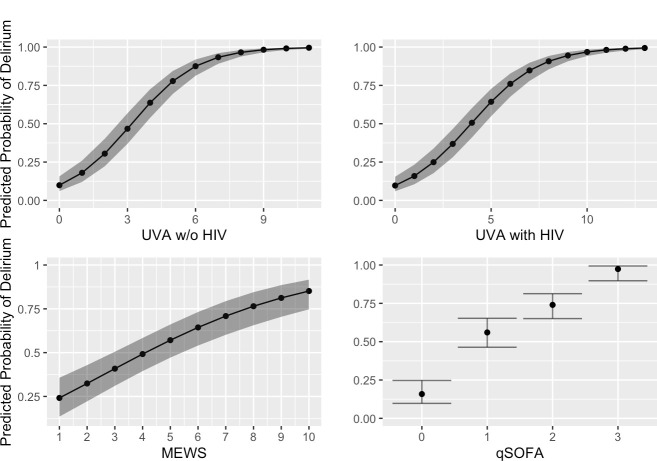
Predicted probability of delirium by severity of illness score at admission.

Among other clinical variables, significant risk factors for delirium included history of stroke (OR 2.70, 95% CI 1.15–7.19), depression/anxiety (OR 1.52, 95% CI 1.08–2.14), and alcohol overuse (OR 1.96, 95% CI 1.39–2.79) (Table 2). All severity of illness scores collected in this study were significant risk factors for delirium, including UVA (OR 1.76, 95% CI 1.61–1.92), UVA without its HIV component (OR 2.00, 95% CI 1.82–2.22), MEWS (OR 1.31, 95% CI 1.21–1.43), and qSOFA (OR 15.12 for qSOFA = 2, 95% CI 8.59–26.63) (**Fig 1**), as were sedatives ordered on admission (OR 3.77, 95% CI 1.70–9.54). Diagnoses involving the neurological organ system (OR 7.66, 95% CI 4.90–12.24), pulmonary organ system (OR 1.91, 95% CI 1.29–2.85), and sepsis (OR 2.44, 95% CI 1.51–4.08) were also significant risk factors. None of the remaining organ system admission diagnosis categories or medical history variables were significant, nor was nutritional status as measured by mid-upper arm circumference. Compared to medical admission, surgical admission was significantly protective against delirium (OR 0.63, 95% CI 0.43–0.90).

This figure shows the probability of delirium (y-axis) by severity of illness score (x-axis) for the UVA score (with and without inclusion of its HIV status variable), MEWS score, and qSOFA score. For UVA, a sigmoidal-shaped association can be seen between increasing UVA score and higher probability of delirium, with an initial rapid increase followed by a leveling off near a delirium probability of 1.0 for the highest UVA score. There appears to be a strong linear association between increasing MEWS and qSOFA scores and higher probability of delirium. In individual regression analyses adjusting for age, sex, and education, all three scores (UVA, MEWS, and qSOFA) were significant risk factors for delirium with P-values < .001 (Table 2). The fitted plot was created using a standardized patient profile derived from the cohort and characterized by age, sex, and education level.

### Combined multivariable regression analysis

**Fig 2:**
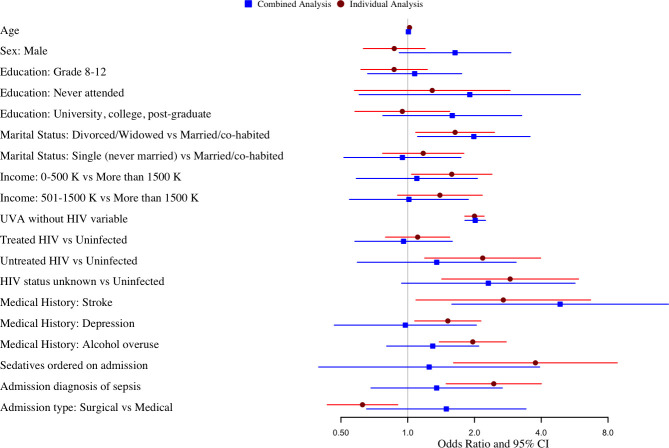
Forest plot comparing results of individual and combined regression analyses.

In combined multivariable regression analysis including significant variables from the individual regression analyses, UVA (OR 2.02, 95% CI 1.82–2.25), history of stroke (OR 4.87, 95% CI 1.64–15.79), and being divorced/widowed (OR 1.99, 95% CI 1.11–3.58) remained significant risk factors for delirium (**Fig 2 and Table 3**). HIV infection and antiretroviral therapy status, monthly income, history of depression/anxiety or alcohol overuse, sedatives ordered on admission, admission type, and sepsis diagnosis were not significant risk factors in the combined analysis.

**Table 3:** Combined multivariable regression analysis^a^.

	Odds Ratio for Delirium	95% CI	P Value
**Age (per year)**	1.01	(0.99–1.02)	.443
**Sex**			
Female	*Reference*		
Male	1.63	(0.92–2.93)	.096
**Education level**			
Grade 1–7	*Reference*		
Grade 8–12	1.07	(0.66–1.76)	.772
Never attended	1.90	(0.60–6.08)	.272
University/post-graduate	1.59	(0.77–3.29)	.209
**Marital status**			
Married/co-habited	*Reference*		
Divorced/widowed	1.99	(1.11–3.58)	.021
Single (never married)	0.95	(0.52–1.74)	.859
**Monthly income (Zambian Kwacha/USD equivalent)**			
More than 1500 Kwacha/150 USD	*Reference*		
501–1500 Kwacha/50.1–150 USD	1.01	(0.55–1.88)	.968
0–500 Kwacha/0-50 USD	1.10	(0.59–2.06)	.768
**Severity of illness**^**b**^			
UVA without its HIV variable	2.02	(1.82–2.25)	< .001
**HIV status and antiretroviral therapy status**			
HIV-uninfected	*Reference*		
HIV-infected with antiretroviral therapy	0.96	(0.58–1.59)	.868
HIV-infected without antiretroviral therapy	1.35	(0.60–3.12)	.474
Unknown HIV status	2.31	(0.96–5.88)	.069
**History of stroke**	4.87	(1.64–15.79)	.006
**History of depression/anxiety**	0.98	(0.46–2.04)	.948
**History of alcohol overuse**	1.30	(0.80–2.09)	.287
**Sedatives ordered on admission**	1.25	(0.42–4.27)	.704
**Sepsis admission diagnosis**	1.35	(0.69–2.70)	.388
**Admission type**			
Medical	*Reference*		
Surgical	1.49	(0.65–3.44)	.343

^a^Significant risk factors from the individual regression analyses were included in a combined multivariable regression analysis. Occupation was excluded due to potential collinearity with income.

^b^The UVA score was chosen *a-priori* as the primary severity of illness score for the analysis. Since HIV is included as a variable in this analysis, we removed the HIV variable from the UVA score.

This figure compares the odds ratios and associated confidence intervals for variables included in both the individual and combined regression analyses. We included the following variables that were found to be significant in the individual regression analyses: marital status, income, UVA severity of illness score (without its HIV variable), HIV status and antiretroviral therapy status, history of stroke, depression/anxiety, alcohol use, sedatives ordered on admission, admission type, and sepsis diagnosis. As the figure demonstrates, the risk factors that remained significant in the combined multivariable analysis were UVA score, being divorced/widowed, and history of stroke, suggesting that these variables are independently predictive of delirium in this setting.

## Discussion

This prospective cohort study identified several risk factors for delirium in a tertiary referral hospital in a resource-limited setting with a high burden of HIV and critical illness. Severity of illness, history of stroke, and being divorced/widowed were significant risk factors for delirium in both the individual and combined regression analyses. To our knowledge, this is the first prospective cohort study conducted in an LMIC designed to rigorously assess risk factors for delirium among general medical and surgical inpatients in a non-ICU setting, using a validated tool for delirium assessment over multiple days of hospital admission.

It is noteworthy that nearly 50% of patients in this acutely ill non-ICU population had delirium at admission, a rate comparable to that of ICU patients in HICs [1]. This rate is substantially higher than prior point-estimates from studies of non-ICU patients in sub-Saharan Africa, which have generally not used validated criteria for delirium assessment [9]. Furthermore, using the same cohort we found that delirium was a strong, independent predictor of 6-month mortality and disability, with a significant dose-response association found between increasing days of delirium and worse clinical outcomes [12]. The high prevalence and poor outcomes of delirium in this patient population emphasize the importance of identifying potential risk factors to improve the prevention, detection, and management of delirium in LMICs.

Severity of illness, as measured by the UVA score, was a powerful and independent predictor of delirium in both the individual and combined regression analyses. With an OR of 2.02 in the combined analysis, each one-point increase in the UVA score independently predicted a two-fold increase in the odds of developing delirium. This is consistent with findings from HICs that increasing severity of illness is an independent risk factor for delirium in ICUs [20], general medical wards [21], and surgical settings [22]. A study of medical inpatients 60 years or older in Tanzania also found severity of illness, as measured by the National Early Warning Score (NEWS), to be a significant delirium risk factor [23].

While the specific mechanisms by which severity of illness affects delirium are still being elucidated, recent research has identified distinct clinical phenotypes of delirium during critical illness, including sedative-associated, hypoxic, and septic delirium [24]. For example, it has been shown that acute hypoxia can cause neurologic injury [25] and systemic inflammation from sepsis can lead to chronic neuroinflammation and neurodegeneration [26]. It is conceivable that the UVA score, which incorporates vital signs data including blood pressure, heart rate, temperature, and oxygen saturation, could be a clinical marker of pathophysiological derangements in severe illness such as hypoxia and systemic inflammation that can cause direct brain injury leading to delirium.

UVA, which was derived and validated using sub-Saharan African hospitalized cohorts, may have utility as a predictive tool for delirium risk assessment, prevention, and early identification in resource-limited settings. For example, a UVA score of 6 was predictive of a greater than 85% probability of having delirium in this cohort. Consistent and standardized use of the UVA score could potentially identify critically ill patients at high risk for delirium, thus allowing medical staff in LMICs to focus delirium assessments on these at-risk patients.

Given that UVA was strongly and independently predictive of delirium in this setting, the high severity of illness at hospital admission in this cohort (mean UVA score of 4.73, indicating a high risk for in-hospital mortality) suggests that severity of illness may be a substantial contributor to the high rates of delirium seen in this patient population (nearly 50% at enrollment). The high burden of both severe illness and delirium at hospital presentation in this socioeconomically and medically vulnerable patient population implies that many of these patients may have been critically ill and delirious before arriving from the community or referring clinics and hospitals due to delayed or inequitable access to acute care. Effective use of and improved access to critical care resources in LMICs has been recognized as an important area of focus for future research [27]. Because there is a strong dose-response association between number of days of delirium and worse clinical outcomes [2–4], it is important to identify delirious patients as quickly as possible. UVA may therefore have additional utility for LMIC health systems as a way to rapidly identify and triage deteriorating patients at high risk of delirium for further assessment and possible transfer for more advanced care. Similar efforts with vital sign-directed therapy have shown promise in resource-limited settings [28].

In this investigation benzodiazepine sedatives ordered on admission were a risk factor for delirium only in the individual analysis but not in the combined analysis, suggesting that sedatives may not be independently predictive of delirium in this setting. This finding is surprising given that sedatives are one of the most well-established modifiable risk factors for delirium in HIC ICUs [29]. However, these results must be interpreted with caution as we were unable to document exactly when admission sedatives were given in relation to the participants’ first delirium assessment. Furthermore, only 4.9% of the cohort had sedatives ordered on admission. These results suggest that sedatives are likely not substantial contributors to the high prevalence of delirium found in this non-ICU patient cohort.

History of stroke was also a risk factor for delirium in the combined analysis, consistent with prior research [30], as was being divorced or widowed, suggesting that these are independent risk factors for delirium in this setting. We do not know if being divorced or widowed is a causal association, but it is conceivable that divorced/widowed patients may seek care at more advanced stages as a result of not having anyone to bring them to the hospital, or may have no one at the hospital bedside to help them remain oriented. This latter point may be of particular importance, as family engagement and empowerment has been recognized as a vital component of ICU delirium prevention [31]. In addition, divorced and widowed persons may have less support and connection to care when they leave the hospital, further increasing their risk of having poor clinical outcomes following severe illness.

HIV and sepsis are significant sources of morbidity and mortality in LMICs [32,33]. Zambia has an HIV prevalence of 11.5%, 45,000 new HIV infections each year, and 14,000 annual deaths due to AIDS [34]. However, HIV and sepsis do not appear to be independent risk factors for delirium in this investigation after adjusting for other covariates that were predictive of delirium in the combined analysis, such as severity of illness. Indeed, the high prevalence of delirium (57.6%) among participants with HIV in this setting may be explained more by the high severity of illness among this cohort rather than HIV infection itself.

The study has several limitations. First, participants were enrolled using a convenience sample approach given the large number of daily hospital admissions. This was addressed through a point prevalence study in the same setting that enrolled all admitted patients during a seven-day period, which found a delirium point prevalence of 43.6%. This was similar to the 47% prevalence found in the primary cohort at enrollment, suggesting that the primary cohort was likely a representative sample of all patients admitted to the hospital. Second, due to the large number of participants being followed each day, staff could only evaluate participants for delirium during the first three days of hospitalization. This limited our ability to evaluate potentially modifiable hospital-based risk factors for delirium. Third, resource limitations precluded laboratory confirmation of suspected TB cases, and so the TB-related variables used in our study (based on either clinical assessment or antituberculosis treatment on or prior to admission) could not conclusively identify active TB infection.

## Conclusions

In conclusion, severity of illness, history of stroke, and being divorced or widowed were independent risk factors for delirium in this cohort study of acutely ill hospitalized patients in Zambia. Further studies are needed to identify potentially modifiable risk factors for delirium, including pre-hospital variables, to inform methods to prevent, detect, and manage delirium and to improve patient outcomes in LMICs.
